# Detection of Usutu virus infection in wild birds in the United Kingdom, 2020

**DOI:** 10.2807/1560-7917.ES.2020.25.41.2001732

**Published:** 2020-10-15

**Authors:** Arran J Folly, Becki Lawson, Fabian ZX Lean, Fiona McCracken, Simon Spiro, Shinto K John, Joseph P Heaver, Katharina Seilern-Moy, Nic Masters, Luis M Hernández-Triana, L Paul Phipps, Alejandro Nuñez, Anthony R Fooks, Andrew A Cunningham, Nicholas Johnson, Lorraine M McElhinney

**Affiliations:** 1 Virology Department, Animal and Plant Health Agency, Surrey, United Kingdom; 2 Institute of Zoology, Zoological Society of London, Regent’s Park, London, United Kingdom; 3 Pathology Department, Animal and Plant Health Agency, Surrey, United Kingdom; 4 Wildlife Health Services, Zoological Society of London, Regent’s Park, London, United Kingdom

**Keywords:** Flaviviridae, Emerging Infectious Diseases, Zoonosis, Vector Borne Disease, Usutu virus, Passerine birds

## Abstract

In August 2020, as part of a long-term disease surveillance programme, Usutu virus was detected in five Eurasian blackbirds (*Turdus merula*) and one house sparrow (*Passer domesticus*) from Greater London, England. This was initially detected by reverse transcription-PCR and was confirmed by virus isolation and by immunohistochemical detection of flavivirus in tissues. Phylogenetic analysis identified Usutu virus African 3.2 lineage, which is prevalent in the Netherlands and Belgium, suggesting a potential incursion from mainland Europe.

In recent years, the zoonotic viruses, West Nile virus (WNV) and Usutu virus (USUV) have spread extensively throughout mainland Europe. Emergence is understood to be facilitated by the movement of wild birds, the vertebrate reservoir, and mosquitoes, the arthropod vector. This presents a risk of virus introduction to the United Kingdom (UK). As a result, dead wild birds in the UK are tested for both viruses during the vector-active season. 

During the late summer of 2020, USUV RNA was detected in six passerine birds found in Greater London. Here we confirm the details of the initial detection and molecular characterisation of the virus.

## Wild bird *Flavivirus* surveillance in the United Kingdom

Usutu virus (family: *Flaviviridae*, genus: *Flavivirus*) is a single-stranded RNA virus that is maintained in a natural enzootic cycle between mosquitoes, which act as vectors, and birds, which are the main amplifying hosts [1]. Epizootics involving large-scale bird die-offs have occurred across mainland Europe, including Belgium and the Netherlands [2,3], but with no evidence of emergence in the United Kingdom (UK). As part of a long-term disease surveillance programme in Great Britain (2005 until present), coordinated by the Institute of Zoology (https://www.gardenwildlifehealth.org) and the Animal and Plant Health Agency, morbidity and mortality in wild birds is recorded and carcasses are submitted for post-mortem examination (PME). Targeted surveillance for WNV and USUV is conducted on samples collected from these wild birds during the active mosquito season, April to November inclusive [4]. Since 2005, samples from more than 2,550 wild birds have been submitted for molecular testing, of which 643 have been screened specifically for USUV.

## Outbreak identification and confirmation

Between 15 July and 26 August 2020, five Eurasian blackbirds (*Turdus merula*) and one house sparrow (*Passer domesticus*) were submitted from a single area in Greater London. PMEs were conducted according to a standardised protocol [5]. The blackbirds comprised three adult males in thin or emaciated condition, based on assessment of muscle condition and body fat reserves, and two juveniles of undetermined sex in normal body condition. Clinical signs had been observed in two of the blackbirds: one was dehydrated and unable to grip, and was therefore euthanised on welfare grounds; the second was found unresponsive and subsequently died. The house sparrow was an adult male in thin body condition that was found dead.

Following PME, total RNA was extracted from brain and kidney samples to screen for USUV, WNV and Sindbis virus (SINV). Neither WNV nor SINV RNA was detected in any of the samples, however, USUV RNA was detected in all samples using a specific reverse transcription PCR (RT-PCR) assay [6] (cycle threshold (Ct) range: 21.94–30.90). Detection of USUV RNA was confirmed using a pan-flavivirus RT-PCR [7] that provided sufficient amplicon (165 bp) for Sanger sequencing. All sequences showed 100% similarity to USUV African 3.2 lineage (GenBank accession number: MN122254), isolated from a blackbird in the Netherlands [2]. We also sequenced the cytochrome oxidase 1 gene to confirm host species identity of tissue samples (Supplementary material). In addition, we have isolated USUV from RT-PCR-positive brain and kidney tissues from all six birds using Vero cells. This has been confirmed by visualisation of cytopathic effect and RT-PCR [6].

Flavivirus envelope (E) antigen was detected by immunohistochemistry on formalin-fixed paraffin-embedded tissue sections from all six birds. Tissue sections were quenched for endogenous peroxidase activity, virus antigens retrieved with proteinase enzyme buffer (DAKO, Glostrup, Denmark), followed by immunolabelling with mouse monoclonal antibody against flavivirus E antigen (ab155882, Abcam, Cambridge, UK; 2 µg/mL) or a concentration-matched mouse IgG class 2a isotype control (ab18415, Abcam, Cambridge, UK). Tissue sections were then incubated with DAKO mouse EnVision + System and horseradish peroxidase (DAKO, Glostrup, Denmark), visualised using 3,3-diaminobenzidine (Sigma Aldrich, Missouri, United States (US)) and counterstained in Mayer’s haematoxylin (Leica, Illinois, US). We used Vero cells infected with Japanese encephalitis virus or WNV as positive controls for flavivirus immunolabelling. Usutu RNA negative birds were used as negative controls (Supplementary material).

**Figure 1:**
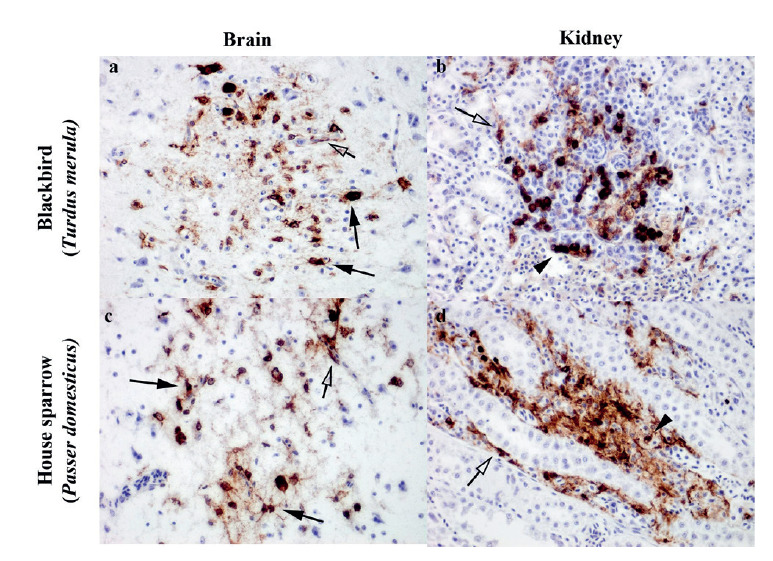
Detection of flavivirus envelope antigen in blackbird (*Turdus merula*) and house sparrow (*Passer domesticus*) using immunohistochemistry on formalin-fixed paraffin-embedded tissue sections, United Kingdom, 2020

Brain and kidney samples were used to confirm presence of virus antigen to corroborate the RT-PCR results. Light microscopy examination of the brain and kidney sections of each of the blackbirds and house sparrow revealed positive immunolabelling for flavivirus E antigens. In the brains of five birds (brain not available for histology from one blackbird), virus antigens were present in the neurons (Figure 1a and, c). In the kidneys of all six birds, presence of virus antigens in renal tubules were associated with mild to moderate multifocal lymphoplasmacytic tubulointerstitial nephritis (Figure 1b and d). In addition, capillaries in the brain and kidney from both passerine species were immunopositive for flavivirus antigens (Figure 1). 

Solid arrow: neurons; non-solid arrow: endothelial cells; solid arrow head: renal tubular epithelial cells. Images taken at 400× magnification.

## Sequence analysis

**Figure 2:**
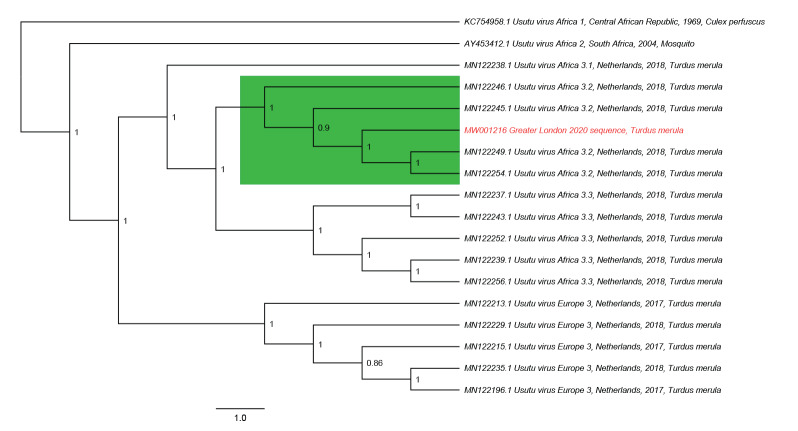
Bayesian phylogenetic tree of a de novo assembled USUV lineage from an infected blackbird (*Turdus merula*), United Kingdom, 2020

Extracted RNA samples were submitted for next generation sequencing (NGS). A Nextera XT DNA library preparation kit (2 × 150 bp reads, Illumina, San Diego, US) was used for library preparation. Sequencing was carried out on an Illumina MiSeq sequencer. We performed a de novo assembly using SPAdes v3.14.1 on one blackbird sample (based on Ct value, see Supplementary material) and used the resulting contig list as a seed for a BLAST search. One contig (10,922 bp) aligned to an USUV African 3.2 isolate (GenBank accession number: MN122254; 99.80% identity). We used the de novo contig to align reads from all six RNA extractions using a combination of Burrows-Wheeler Aligner v0.7.13 and SAMtools v1.9. Read alignment and genome coverage for all sequences was inspected in Tablet v1.19.09.03 (Supplementary material). Following consensus construction, the de novo assembled Greater London 2020 sequence (GenBank accession number: MW001216) was aligned against 17 USUV GenBank sequences (Supplementary material) in Mafft v7.471. The alignment was imported into BEAST v1.10.4 and used to construct a Bayesian phylogenetic tree using the GTR + I + G nucleotide substitution model and 10,000,000 Markov chain Monte Carlo generations (Figure 2). Log files were analysed in Tracer v1.7.1 to check effective sample size and a 10% burn-in was included (TreeAnnotator v1.10.4), before tree visualisation and annotation in FigTree v1.4.4. The Greater London 2020 sequence formed a distinct, well supported clade with African 3.2 lineages of USUV. The NGS reads failed to align to a European WNV genome (GenBank accession number: MH924836).

USUV: Usutu virus.

The tree is based on 10,922bp of coding sequence from the USUV genome. The Greater London 2020 sequence (highlighted in red) forms a distinct clade with Africa 3.2 lineages previously detected in the Netherlands (highlighted in green). Node labels represent posterior probabilities and scale bar represents substitutions per site. Accession number, country, year of detection and host species are included for each sequence.

## Discussion

Here we present data to support the emergence of USUV in wild birds in the UK in 2020. Our results show that an African 3.2 lineage of USUV has infected two species of wild birds in Greater London. Given that mortality occurred over a 5-week period, it is likely that autochthonous transmission of USUV in local passerine populations has occurred, probably vectored by indigenous mosquitoes [1]. While there have been previous reports of USUV neutralising antibodies from birds in the UK, the authors did not report detecting virus using either virus isolation or molecular methods [8,9]. In addition, targeted wild bird disease surveillance for USUV since 2005 has not detected earlier virus incursions [4]. However, it is important to note that our detection is unlikely to represent the incursion event, which may have occurred at an earlier date and at another location where emergence may have been facilitated by migratory birds or transported mosquitoes [2]. The average temperature in the late spring and early summer of 2020 in the UK was 1–2 °C higher than average [10], which may have been permissive for USUV replication and subsequent transmission by mosquito vectors, allowing its establishment in native wild bird populations. Mosquito surveillance, in collaboration with Public Health England, is being conducted in the location where the infected birds were found, to ascertain whether USUV is circulating in local vectors. On 30 September 2020, mosquitoes were active at the index site and therefore, if climatic conditions are permissible, transmission of USUV to wild birds may be ongoing.

The USUV Africa 3.2 lineage is widespread in mainland Europe and presents a likely source population for the identified UK outbreak [2]. Originally isolated in South Africa in 1959, USUV has since emerged across mainland Europe, following a similar pattern to the closely related WNV [11]. There are 10 recognised USUV lineages co-circulating in Europe, and this is likely to be a result of independent introduction events [12]. Consequently, other lineages of USUV may emerge in the UK. 

*Turdus merula* and *P. domesticus* are susceptible to USUV and can develop systemic infections which our findings corroborate [13-17]. Indeed, infection with USUV has caused morbidity and mortality across Europe in passerines, especially *T. merula*, sometimes on a scale sufficient to cause population declines [18]. Although infection in humans is rare, USUV is a zoonosis that is predominantly asymptomatic but can result in neurological disease [19,20]. In addition, the virus can also be transmitted by blood transfusion, highlighting the importance for USUV screening of blood products as a preventative control measure [21,22]. In light of our findings, the UK government’s Human Animal Infections and Risk Surveillance group (HAIRS) has revised and increased its public health risk assessment for USUV, to a low probability of transmission and a low to moderate impact [23]. In addition, the HAIRS report recommends that the Standing Advisory Committee on Transfusion Transmitted Infections, the Advisory Committee on Dangerous Pathogens, and the UK Zoonosis Network are to be advised of the revised public health risk within the UK [23]. The detection of USUV in the UK has implications for both animal and public health and future outbreaks in wild birds may occur and should be monitored.

## Supplementary Data

611000 Supplement
