# Supplementary material for: Detection of Usutu virus infection in wild birds in the United Kingdom, 2020
Source: Euro Surveill. 2020 Oct 15;25(41):2001732. doi: 10.2807/1560-7917.ES.2020.25.41.2001732 (PMC7565854; doi:10.2807/1560-7917.ES.2020.25.41.2001732)
Supplement: Supplement [file 20-01732_FOLLY_Supplement.pdf]

Supplementary Information

This supplementary material is hosted by Eurosurveillance as supporting information alongside the article Detection of Usutu virus infection in wild birds in the United Kingdom, 2020, on behalf of the authors, who remain responsible for the accuracy and appropriateness of the content. The same standards for ethics, copyright, attributions and permissions as for the article apply. Supplements are not edited by Eurosurveillance and the journal is not responsible for the maintenance of any links or email addresses provided therein

COI DNA Barcoding methodology to determine tissue provenance

Residual genomic DNA present in the RNA extraction was used to amplify the *COI* barcoding region gene to support the species morphological identification. The COI barcoding region was amplified using the primers LCOI490 and HCO2198 (Folmer *et al*., 1994). PCR reactions were performed in a total volume of 50 µL (Hernandez-Triana *et al*., 2017) using 2 µL of genomic DNA, 1 X NH_4 buffer, 2 pmol/µL dNTPs, 1.5 mM $MgCl_2$, 10 pmol/µL of each primer, 0.6U *Taq* DNA polymerase (Bioline, UK) and 20 mg/mL bovine serum albumin. The thermal profile consisted of 1-min initial cycle at 94°C followed by a pre-amplification 5 cycles of 94°C for 1 min, 45°C for 1.5 min, 72°C for 1.5 min, and an amplification step of 35 cycles of 94°C for 1 min, 57°C for 1.5min, 72°C for 1.5 min with a final extension of 72°C for 5 min. PCR products were separated by electrophoresis in 1.5% agarose gel and samples showing the expected band size were purified using the QIAquick PCR purification kit (Qiagen, UK) and sequenced in both directions using the ABI PRISM® BigDye® Terminator sequencing kit (Applied Biosystems, UK) following the manufacturer´s instructions. All sequences were assigned to a species when agreement was ≥96% with sequences of named species in GenBank. Tissue samples 1-5, described as blackbird and tissue sample 6 described as a house sparrow were confirmed using this *COI* method.

References:

Folmer, O., Black, M., Hoeh, W., Lutz, R., Vrijenhoek, R. (1994) DNA primers for amplification of mitochondrial cytochrome c oxidase subunit I from diverse metazoan invertebrates.*Molecular Marine Biology and Biotechnology*. **3**: 294–299.

Hernández-Triana, L.M., Brugman, V.A., Prosser, S.W.J., Weland, C., Nikilova, N., Thorne, L., de Marco, M.M.F., Fooks, A.R. & Johnson, N. (2017) Molecular approaches for blood meal analysis and species identification of mosquitoes (Insecta: Diptera: Culicidae) in rural locations in southern England, United Kingdom. *Zootaxa*, **4250**:067-076.

Negative immunohistochemistry slides for a RT-PCR negative brain and kidney sections from a blackbird and house sparrow

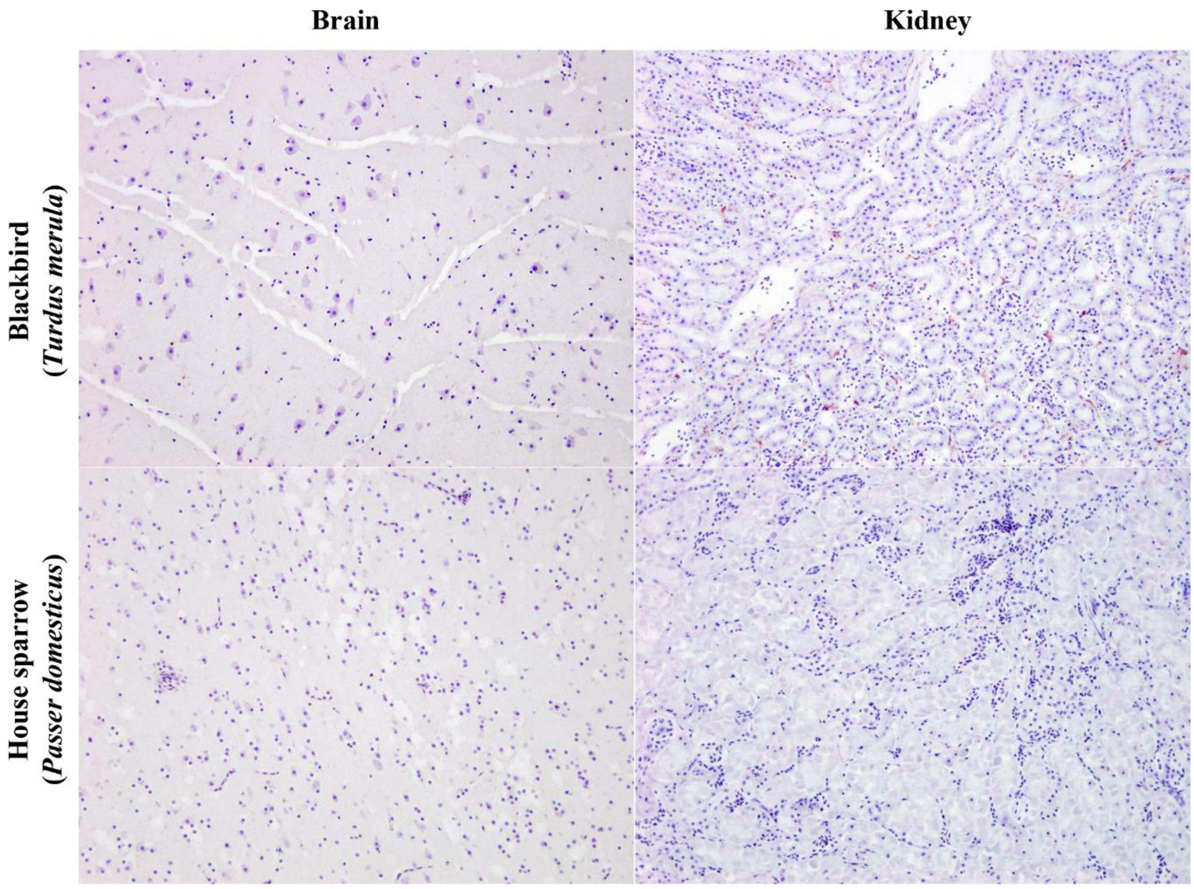

Figure 1. Immunohistochemical labelling of formalin-fixed paraffin-embedded tissue sections obtained from USUV RT-PCR negative blackbird (*Turdus merula*) and house sparrow (*Passer domesticus*). Images taken at 200x magnification.

Phylogenetic analysis and Genbank sequence provenance

Table 1. Accession numbers used for alignment against Greater London 2020 sequence (accession number MW001216) and subsequent phylogenetic analysis.

Accession number	Length (bp)	Representative lineage
KC754958	10745	Africa 1
AY453412	11064	Africa 2
MN122238	10932	Africa 3.1
MN122245	10932	Africa 3.2
MN122246	10932	Africa 3.2
MN122249	10932	Africa 3.2
MN122254	10932	Africa 3.2
MN122237	10932	Africa 3.3
MN122239	10932	Africa 3.3
MN122242	10932	Africa 3.3
MN122252	10932	Africa 3.3
MN122256	10932	Africa 3.3
MN122196	10932	Europe 3
MN122213	10932	Europe 3
MN122215	10932	Europe 3
MN122229	10932	Europe 3
MN122235	10932	Europe 3

Consensus sequence read coverage depths aligned to a *de novo* assembled contig that matched an Africa 3.2 lineage of Usutu virus.

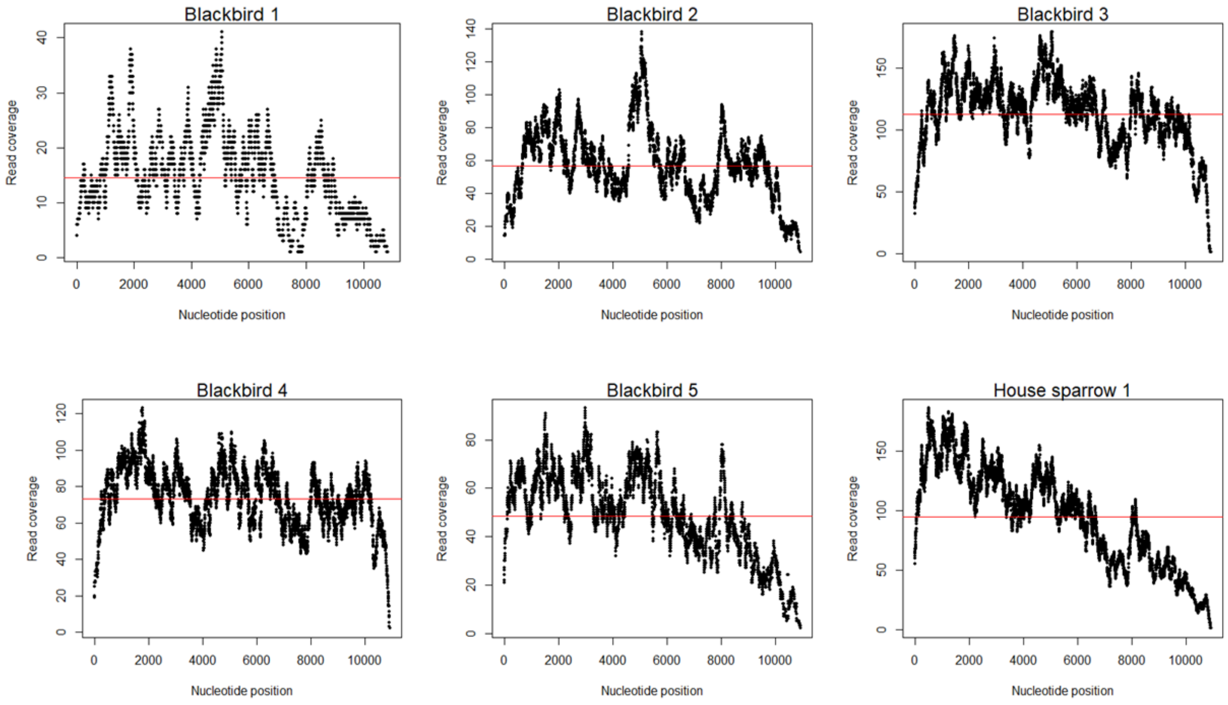

Figure 2. Read coverage plots for consensus sequences, aligned to a *de novo* contiguous sequence (10,922 bp), for five blackbird and one house sparrow RNA samples. The average coverage depth is marked with a red line.

Additional references for NGS analysis /phylogenetic packages and diagnostic PCRs

SPAdes= Bankevich, A., Nurk, S., Antipov, D., Gurevich, A.A., Dvorkin, M., Kulikov, A.S., Lesin, V.M., Nikelenko, S.I., Pham, S., Prjibelski, A.D., Pyshkin, A.V., Sirotkin, A.V., Vyahhi, N., Tesler, G., Alekseyev, M.A., Pevzner, P.A. (2012) SPAdes: A new genome assembly algorithm and its applications to single cell sequencing. *Journal of Computational Biology*. **19**: 455-477.

BWA = Li, H., Durbin, R. (2009) Fast and accurate short read alignment with Burrows-Wheeler transform. *Bioinformatics*. **25**: 1754-1760.

SAMtools = Li, H., Handsaker, B., Wysoker, A., Fennell, T., Ruan, J., Homer, N., Marth, G., Abecasis, G., Durbin, R. (2009) The sequence alignment/Map format and SAMtools. *Bioinformatics*. **25**: 2078-2079.

Tablet = Milne, I., Stephen, G., Bayer, M., Cock, P.J., Pritchard, L., Cardle, L., Shaw, P.D., Marshall, D. (2013) Using Tablet for visual exploration of second-generation sequencing data. *Briefings in Bioinformatics*. **14**:193-202.

Mafft = K., Katoh, Misawa, K., Kuma, K., Miyata, T. (2002) MAFFT: a novel method for rapid multiple sequence alignment based on fast Fourier transform. *Nucleic Acid Research*. **30**: 3059-3066.

BEAST = Suchard, M.A., Lemey, P., Baele, G., Ayres, D.L., Drummond, A.J., Rambaut, A. (2018) Bayesian phylogenetic and phylogenomic data integration using BEAST 1.10. *Virus Evolution*. **4**: vey016

Tracer = Rambaut, A., Drummond, A.J., Xie, D., Baele, G., Suchard, M.A. (2018) Posterior summarisation in Bayesian phylogenetics using tracer 1.7. *Systematic Biology*. **67**: 901-904.

FigTree = Rambaut, A. (2014) Figtree v1.4.2 A graphical viewer of phylogenetic trees. Available from (http://tree.bio.ed.ac.uk/software/figtree/)
